# Detection of Abundant Non-Haematopoietic Circulating Cancer-Related Cells in Patients with Advanced Epithelial Ovarian Cancer

**DOI:** 10.3390/cells8070732

**Published:** 2019-07-17

**Authors:** Juhi Kumar, Dimple Chudasama, Charlotte Roberts, Mikael Kubista, Robert Sjöback, Jayanta Chatterjee, Vladimir Anikin, Emmanouil Karteris, Marcia Hall

**Affiliations:** 1 Department Life Sciences, Brunel University London, Uxbridge UB8 3PH, UK; 2 Mount Vernon Cancer Centre, Middlesex HA6 2RN, UK; 3 TATAA Biocenter, 411 03 Göteborg, Sweden; 4 Laboratory of Gene Expression, Institute of Biotechnology CAS, v.v.i., 252 50 Vestec, Czech Republic; 5 Faculty of Health and Medical Sciences, School of Biosciences and Medicine, University of Surrey, Guildford, Surrey GU2 7XH, UK; 6 Division of Thoracic Surgery, The Royal Brompton & Harefield NHS Foundation Trust, Harefield Hospital, London UB9 6JH, UK; 7 Department of Oncology and Reconstructive Surgery, Sechenov First Moscow State Medical University, 119146 Moscow, Russia

**Keywords:** advanced epithelial ovarian cancer, circulating tumour cells, circulating endothelial cells

## Abstract

**Background**: Current diagnosis and staging of advanced epithelial ovarian cancer (aEOC) has important limitations and better biomarkers are needed. We investigate the performance of non-haematopoietic circulating cells (CCs) at the time of disease presentation and relapse. **Methods**: Venous blood was collected prospectively from 37 aEOC patients and 39 volunteers. CCs were evaluated using ImageStream Technology™ and specific antibodies to differentiate epithelial cells from haematopoetic cells. qRT-PCR from whole blood of relapsed aEOC patients was carried out for biomarker discovery. **Results**: Significant numbers of CCs (CK^+^/WT1^+^/CD45^−^) were identified, quantified and characterised from aEOC patients compared to volunteers. CCs are abundant in women with newly diagnosed aEOC, prior to any treatment. Evaluation of RNA from the CCs in relapsed aEOC patients (n = 5) against a 79-gene panel revealed several differentially expressed genes compared to volunteers (n = 14). Size differentiation of CCs versus CD45^+^ haematopoietic cells was not reliable. **Conclusion**: CCs of non-haematopoetic origin are prevalent, particularly in patients with newly diagnosed aEOC. Exploiting a CC-rich population in aEOC patients offers insights into a part of the circulating microenvironment.

## 1. Introduction

Detection of potentially small populations of non-haematopoetic circulating tumour cells (CTCs) within whole blood in cancer patients represents a significant technical challenge [1,2]. Isolating CTCs is generally attempted by using physical features such as size and enrichment techniques to confirm the identity of epithelial rather than haematopoietic cells. This has relied on surface proteins such as the Epithelial Cell Adhesion Molecule (EpCAM) to confirm/refute their origin as cancer cells.

The most widely used method for CTC analysis is the CELLSEARCH^®^ CTC Test, FDA approved in breast, colorectal and prostate cancer, where identification of CTCs broadly rely on EpCAM, despite evidence of considerable heterogeneity of EpCAM expression during the cell cycle, in different cell lines and a lack of EpCAM in cells undergoing epithelial-mesenchymal transition (EMT) [3,4,5]. The CellSearch test has been explored in patients with ovarian cancer in multiple publications with very low yields (e.g., single CTCs in 7.5 mL of whole blood, which may contain 75 million leucocytes and 50 billion erythrocytes) and is thus of limited utility [6] even in patients with recently diagnosed advanced epithelial ovarian cancer (aEOC) prior to any treatment. Most groups have used EpCAM as the common key target/antigen for the identification and confirmation of epithelial origin, with or without numerous other epithelial markers such as MUC1, MUC16, CK8, CK18, CK19, CA125, DPP4, ERCC1, HER2, MOC-31, PPIC. In clinical practice, immunohistochemistry assays on tissue likely to be of ovarian/fallopian tube/primary peritoneal origin utilise different antigens such as CK7, CK20 and WT1 to confirm the diagnosis of high-grade serous cancer prior to definitive treatment. We sought to explore the use of these antigens to identify CCs in aEOC patients.

The primary aim of this study was to examine whole blood of patients with aEOC using ImageStream™ (Amnis), a high-definition circulating cell (HD-CC) imaging flow cytometry system, without using surface protein-based enrichment techniques. This technology allows multiple antibody applications and presents CCs in sufficient definition to satisfy diagnostic pathology image quality requirements [7,8]. Clinical immunohistochemistry cell surface and nuclear immunohistochemical antibody markers, initially pan-cytokeratin (PCK; AE1/AE3), CD45, and DRAQ5™ were used to identify and characterise non-haematopoetic and haematopoetic cells. Subsequently WT1 antibody was used to specifically identify cells of high grade serous (ovarian, fallopian tube/primary peritoneal) origin. Correlations were made with serum CA125 levels during treatment and CC levels in 4 patients. We have expanded on our observations, to include further interrogation of some liquid biopsies by means of gene expression from whole blood.

## 2. Materials and Methods

### 2.1. Cell Culture

SKOV3 and MDAH-2774 ovarian cancer cells (ATCC) were grown in DMEM (Dulbecco’s Modified Eagle’s Medium, Gibco, Paisley, UK) supplemented with 10% FBS (fetal bovine serum, Gibco), 1% penicillin/streptomycin (Gibco) and 1% L-glutamine (Gibco); at 37 °C/5% CO_2_. PEO1 cells were grown in RPMI-1640, 10% FBS, 1% penicillin/streptomycin at 37 °C/5% CO_2_.

Healthy volunteer (control) whole blood (1mL) was spiked individually with 2 cell lines, SKOV3 and MDAH-2774, to mimic liquid biopsies that were processed as before and analysed using the enumeration features of the ImageStream™ based on size criteria and staining for both EpCAM and pan-cytokeratin (AE1/AE3) antibodies. This procedure was repeated with the introduction of different quantities of SKOV3 and MDAH-2774 cells, to assess the efficiency of cell retrieval and loss when using EpCAM and AE1/AE3 markers. The cell suspension was resuspended in different volumes to give a variety of cell concentrations between 20–200,000 cells/mL and spiked in 1mL blood from a donor.

### 2.2. Patients

Patients and healthy volunteers included in this report were enrolled in the prospective CICATRIx clinical study where blood samples are collected from healthy volunteers and patients with advanced cancer attending Mount Vernon Cancer Centre (East and North Hertfordshire NHS Trust) for exploratory biomarkers. All volunteers and patients provided written informed consent for participation in the study and for use of their donated tissue (patients only) and blood specimens.

The CICATRIx study: Sample collection study to explore circulating tumour cells, cell free DNA and leucocytes with ImageStream analysis in patients with various cancers. Protocol number RD2016-08 was approved by the West Midlands–South Birmingham Ethics Committee (reference 16/WM/0196).

TRANS-METROBIBF: Sample collection study to investigate the predictive role of Circulating Tumour Cells (CTCs) and their gene expression in relation to outcome in multiply-relapsed ovarian cancer patients. Protocol number: RD2013-01 was approved by the South East Coast–Surrey Ethics Committee (reference 14/LO/0792).

The studies were performed in accordance with the Declaration of Helsinki.

5–10 mL of whole blood was taken from patients at regular intervals with a tissue diagnosis of high-grade serous ovarian cancer (HGSOC), in one of the three groups:

(1) Relapse—patients whose aEOC had relapsed following prior remission, and who required further chemotherapy treatment.

(2) NACT—patients whose primary treatment at diagnosis was Neo-Adjuvant Chemotherapy.

(3) post-PDS—patients who had had Primary Debulking Surgery as their first treatment for aEOC.

39 healthy female volunteers donated 5–10 mL whole blood, which was also screened for cells that would stain with pan-cytokeratin and WT1 antibodies. Whole blood samples were assayed within 6 days of venesection.

### 2.3. Clinical Cohorts

**Figure 1:**
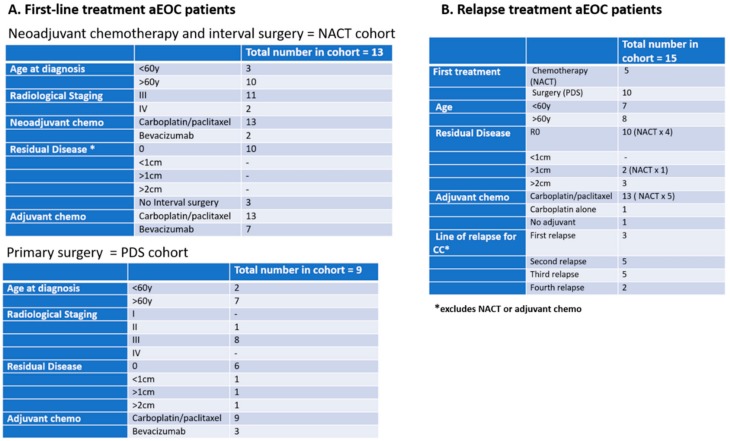
Details of patients enlisted in the study. (**A**) Neoadjuvant chemotherapy and interval surgery; NACT cohort (n = 13) and Primary surgery, PDS cohort (n = 9); (**B**) Relapse treatment aEOC patients (n = 15).

Three cohorts of patients with aEOC were included in this exploration of CCs (Figure 1):

(1) In the Relapse cohort, patient samples were taken prior to the first infusion of the course of chemotherapy and at the time of subsequent infusions. This group included 5 patients who participated in the METRO-BIBF clinical trial, a phase II, randomised, placebo-controlled, multicentre, feasibility study of low dose (metronomic) cyclophosphamide with and without nintedanib (BIBF 1120)/placebo (all patients received oral cyclophosphamide). Bloods tests were performed every 6 weeks at clinic review in the METRO-BIBF patients and their samples assayed for pan-cytokeratin, DRAQ5™ and CD45, but not WT1 expression. All other relapsed patients had WT1 expression examined.

(2) In the NACT cohort, patient samples were taken following confirmed histological diagnosis and prior to the start of their chemotherapy and at each subsequent treatment. Many of these patients underwent interval surgery following 3–4 chemotherapy cycles.

(3) In the post-PDS cohort, patient samples were taken after surgery and prior to any adjuvant chemotherapy and at each subsequent chemotherapy cycle.

Initially, a comparative assessment was made of a single sample, 30 mL from each of four patients, decanted into 6–7 individual tubes of different types: EDTA, Cell-Free DNA Blood Collection Tube (Streck), PAXgene Blood DNA Tube (Qiagen), and Cell-Free DNA Collection Tube (Roche) to establish the best vehicle for cell preservation prior to assay. Samples were analysed within 4 h of venesection for Day 1 and then daily for a further 6 days to assess the preservation ability of each type of collection tube. Subsequent patient and “control” samples from volunteers were collected in Roche tubes and assayed within 6 days of venesection.

### 2.4. CA125 Measurements

Serum CA125 assays were done for all aEOC patients, as part of standard practice, i.e., approximately each time they had chemotherapy treatment.

### 2.5. Preparing Blood Samples for Imagestream™

One ml of whole blood from each patient was decanted from Roche tubes into a 15 mL Falcon tube and mixed with 9 mL of red blood cell (RBC) lysis buffer (G Biosciences), inverted 10 times and incubated for 10 min with gentle agitation. The solution was then centrifuged at 2500 RPM for 10 min, the supernatant removed and a further 3 mL of RBC lysis buffer added to resuspend the pellet, before being incubated for 10 min at room temperature with gentle agitation. The solution was spun for a further 10 min at 2500 RPM, and the supernatant was aspirated. The pellet was then washed in 1.5 mL of PBS, and the resulting suspension was moved to a 1.5 mL microcentrifuge tube and spun at 3600 RPM for 3 min. Samples were then fixed immediately.

### 2.6. Fixing Cells

Cell pellets were transferred to a 1.5 mL microcentrifuge tube and resuspended in 1mL of ice cold 4% paraformaldehyde (PFA, Sigma, Gillingham, UK) for 7 min. The cell suspension was centrifuged for 2 min at 3600 RPM and the PFA was removed. Cells were washed in prewarmed PBS twice, centrifuging for 2 min at 3600 RPM between each wash. After the cells were fixed and washed with PBS, the pellet was suspended in 0.5% Triton X in PBS (v/v) and incubated on ice for 10 min, followed by centrifugation at 3600 RPM for 3 min to enable membrane permeabilisation for cytoplasmic/nuclear staining.

### 2.7. Staining Cells

Cells were incubated in blocking buffer (10% fetal bovine serum, Gibco, in PBS) for 1 h with gentle agitation, centrifuged for 3 min at 3600 RPM and the blocking buffer was removed. The cells were then incubated either in conjugated antibodies or in appropriate primary antibodies (i.e., WT1, AE1/AE3, CD45, CD34) with blocking buffer overnight at 4 °C with gentle agitation. Following primary antibody incubation, cells were centrifuged for 3 min at 3600 RPM and antibody aspirated. The cells were washed in PBS (Gibco) tween (0.2%) to remove any remaining antibody and centrifuged again for 3 min at 3600 RPM. In case of EpCAM, PBS was removed, and the cells were incubated in secondary antibody (Alexa Fluor 488 anti-mouse) diluted in blocking buffer (1:1000 v/v) for 1 h with gentle agitation. From this step onwards, the cells were protected from light as the fluorophore conjugated to the secondary antibody is light sensitive. Further centrifugation for 3 min at 3600 RPM was undertaken and the secondary antibody was removed. Following a wash in PBS tween and centrifugation, the PBS was removed, and the cells were resuspended in 99 μL Accumax (Innovative Cell Technologies, San Diego, CA, USA) to dissociate any cellular aggregates. 1 μL of DRAQ5™ (Biostatus Ltd. Loughborough, UK) nuclear stain was added before visualisation on the ImageStream. All the data files were then analysed on the IDEAS software [9].

For the differential expression of AE1/AE3 and WT1 in SKOV3 and PEO1 cells, they were visualised under a Leica DM4000 microscope, x60.

### 2.8. Imagestream™

To positively identify ovarian cancer CCs, pan-cytokeratin (AE1/AE3), CD45 (to exclude lymphocytes), WT1 (a nuclear/cytoplasmic stain specific to high grade serous ovarian/fallopian tube/primary peritoneal cancer) and DRAQ5™ a general nuclear stain was all applied in different channels. EpCAM was applied to a subset of patients’ CCs to assess the value of EpCAM-related enumeration. CCs were identified and quantified using the IDEAs software.

### 2.9. RNA Extraction/cDNA Synthesis

If RNA extraction was not performed immediately, 0.5 mL of whole blood was added to 1.5 mL of RNAlater^®^ (Life Technologies, Paisley, UK) in an Eppendorf tube, left at room temperature for up to 72 h and stored at −80 °C until further use. Samples stored in RNALater were first defrosted from −20 °C at room temperature then centrifuged at 13,000 RPM for 1 min, followed by aspirating supernatant. RNA from blood samples were extracted using the Ribopure RNA extraction kit (Ambion, Fisher, UK) according to the manufacturer’s protocol. cDNA concentration was normalised using RNA concentrations determined using the NanoDrop (Thermo Scientific, Waltham, MA, USA).

### 2.10. qPCR TATAA

The CTC GrandPerformance panel (TATAA Biocenter, Göteborg, Sweden) was used for the quantification of ovarian cancer-associated gene expression using reverse transcription quantitative real-time PCR (qPCR). The isolated mRNA was reverse-transcribed into cDNA using GrandScript (TATAA Biocenter), pre-amplified in a multiplex-PCR using GrandMaster PreAmp (TATAA Biocenter) and quantified by single-plex qPCR using GrandMaster mix (TATAA Biocenter). Data analysis and classification of samples was done with the GenEx software (MultiD).

### 2.11. Statistical Analysis

All statistical tests were performed using GraphPad Prism^®^ Software (GraphPad Software). Statistical analyses were performed using one-way ANOVA followed by Tukey’s Multiple Comparison Test or student’s t-test with significance determined at the level of *p* < 0.05. P values are indicated in graphs as follows; * *p* = 0.01–0.05, ** *p* = 0.001–0.009, and *** *p* < 0.0009.

## 3. Results

### 3.1. Identification of Ovarian Cancer Cells Mixed with Blood In Reconstruction Experiments Using AE1/AE3 and WT1

Initial in vitro experiments were undertaken to ascertain staining of SKOV3 and MDAH274 ovarian cancer cell lines with AE1/AE3 (CK^+^ antibodies), used widely in NHS histopathology laboratories for diagnosis [10]; as well as WT-1, which is an ovarian-specific stain (Figure S1A). Intensity of staining for AE1/AE3 confirmed the ability to differentiate cancer cells from white blood cells when SKOV3 and MDAH-2774 cells were spiked into 1 ml of healthy donor’s blood (Figure S1B). This procedure was repeated with the introduction of different quantities of SKOV3 and MDAH-2774 cells to assess the efficiency of cell retrieval and loss when using AE1/AE3 and EpCAM. Table S1C shows a significantly reduced number of cultured SKOV3 and MDAH-2774 cells identified using EpCAM antibodies compared with AE1/AE3 (CK^+^).

Furthermore, to elucidate any differences in the staining patterns between epithelial and mesenchymal phenotypes, we stained SKOV3 cells (exhibiting an intermediate mesenchymal (IM) phenotype) and PEO1 cells (exhibiting an epithelial (E) phenotype) with CK^+^ and WT1. We measured 100 cells under the microscope, and all cells (100/100) stained positive for WT1. However, 88/100 of PEO1 (E) cells were stained CK^+^, whereas 41/100 SKOV3 (IM) were stained positive of CK at almost a 1:2 ratio (Figure S2).

### 3.2. Validation of Blood Collection Tubes for CC Integrity

**Figure 2:**
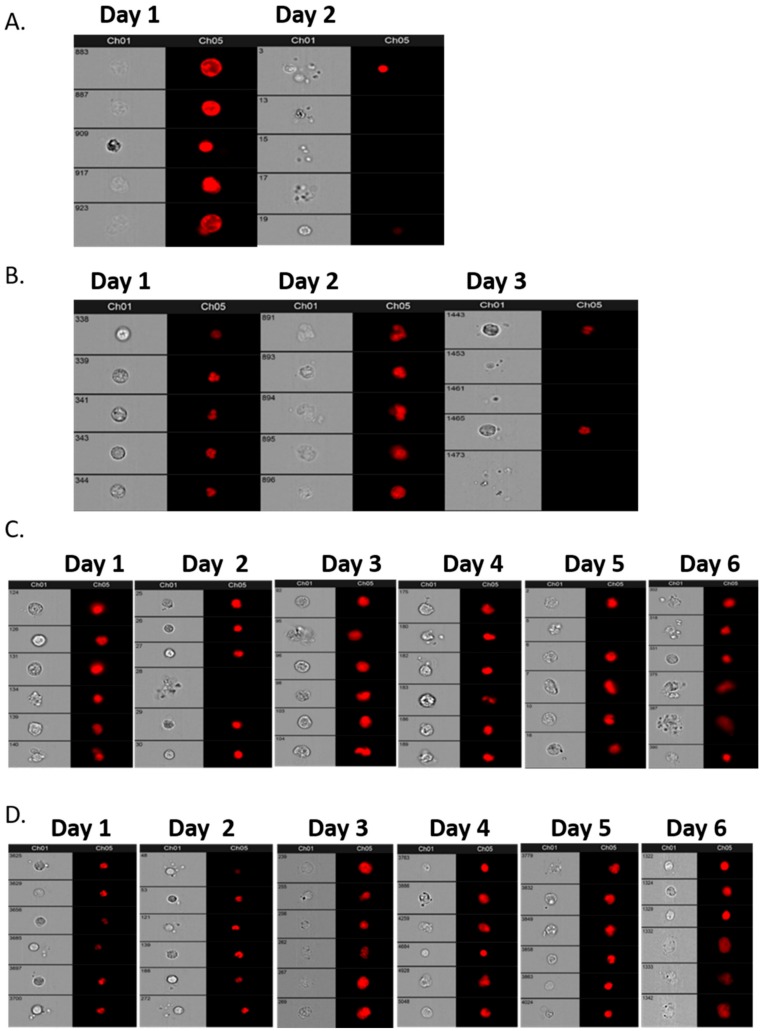
Circulating cell (CC) integrity over 6 days in EDTA tubes (**A**; 2 days), Streck tubes (**B**; 3 days), PAXgene tubes (**C**, 6 days) and Roche (**D**, 6 days) as assessed by Imagestream™. Chanel 1: brightfield, Channel 5: DRAQ5™ nuclear staining (red).

Figure 2 details brightfield microscopy and nuclear definition (using DRAQ5™) of cells from aEOC NACT patient blood samples taken on Day 1 and analysed within 4 h, and then at later time points (days 2–6) to assess the quality of cell preservation for the following tubes: EDTA, Cell-Free DNA Blood Collection Tube (Streck), PAXgene Blood DNA Tube (Qiagen) and Cell-Free DNA Collection Tube (Roche). The Roche was followed by PAXgene tubes preserved CCs for 6 days with reasonable morphology and reliable, reproducible nuclear staining. These tubes were used for all subsequent patient samples (Figure 2A–D).

### 3.3. Expression of AE1/AE3 (CK^+^), WT1, and CD45 in Enriched Blood Samples of Ovarian Cancer Patients

**Figure 3:**
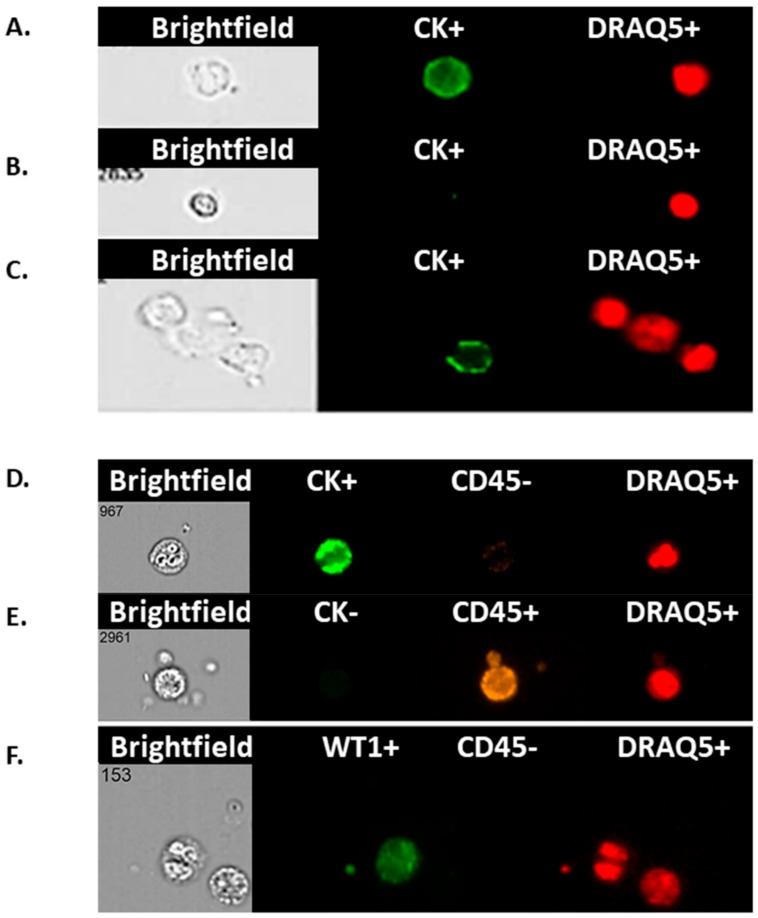
Circulating cells from an ovarian cancer patient blood sample based on staining in a scatter image generated by the Imagestream™. The micrograph shows images of single cells from ovarian cancer patients with: (**A**): positive staining for CK and nuclear staining (DRAQ5) identifying a potential circulating ovarian cell (CC), (**B**): negative staining for CK but positive for DRAQ5 identifying a potential white blood cell (WBC), (**C**): combination of 2 potential WBCs (CK^−^) with a circulating ovarian CC (CK^+^); all three were stained positive for DRAQ5, (**D**): positive staining for CK, negative for CD45 and nuclear staining (DRAQ5) identifying a CC, (**E**): negative staining for CK, positive for CD45 and nuclear staining (DRAQ5) identifying a WBC, (**F**): a combination of 2 cells; one WT1 positive and one negative, both negative for CD45, but positive for nuclear staining (DRAQ5) identifying two potentially different CCs, but not WBCs.

Enriched blood samples were subjected to staining with AE1/AE3 (CK^+^), WT1, and CD45 to differentiate between ovarian CCs and WBCs. Examples include CK^+^, CK^−^ and DRAQ5™^+^ (Figure 3A–C); CK^+^ CD45^−,^ DRAQ5™^+^ CCs (Figure 3D) in comparison with a CK^−^ CD45^+^ DRAQ5™^+^ adjacent white blood cell (Figure 3E). Finally, CCs were also characterised using a WT1^+^, CD45^−^ and, DRAQ5™^+^ staining (Figure 3F).

**Figure 4:**
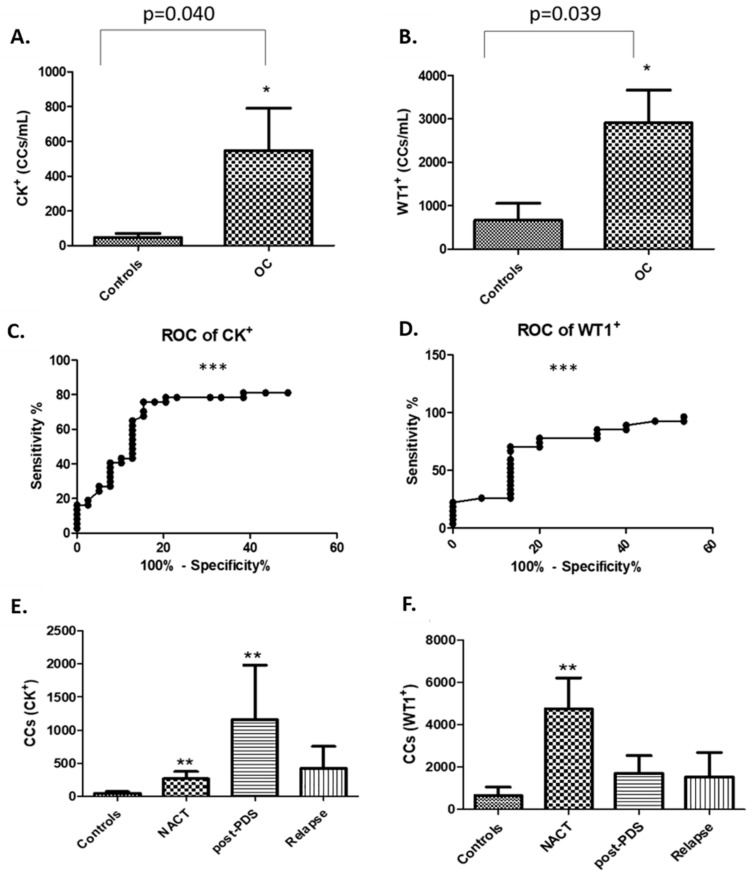
Enumeration of CCs in controls against ovarian cancer (OC) patients: (**A**): CK^+^; * *p* < 0.05, and (**B**) WT1^+^, * *p* < 0.05. ROC curve analysis was used to measure sensitivity and specificity, an AUC of 0.78 (*** *p* < 0.0001) was calculated for CK^+^ (**C**), and an AUC of 0.82 (*** *p* = 0.0006) was calculated for WT1^+^ (**D**). Enumeration of CCs in OC patients prior to any treatment at all (NACT), following primary surgery only (post-PDS), or relapse and comparison to healthy controls. (**E**): CK^+^/CD45^−^/DRAQ5™^+^ and (**F**) WT1^+^/CD45^−^/DRAQ5™^+^, ** *p* < 0.001.

Upon enumeration based on CK^+^, CD45^−^ and DRAQ5™^+^, there was a significant increase in CCs in aEOC patients (n = 37; mean 547 CCs/mL of blood) in comparison with the number of cells staining negative for CK from whole blood from healthy female volunteers (n = 39, mean 47 CCs/mL of blood, Figure 4A; *p* = 0.040).

A similar picture emerged, although with higher actual quantities, when CCs from HGSOC patients were selected based on WT1^+^, CD45^−^ and DRAQ5™^+^ (n = 27, mean 2914) to healthy controls (n = 15, mean 667, Figure 4B; *p* = 0.039). ROC curves for all three measurements were significant, suggesting that this is of potential diagnostic value (Figure 4C,D). We have also tried to correlate these findings with histopathological immunostaining data; from 22 (out of 29) reports for the WT1^+^ patients and 15 reports for the CK^+^ patients. In those reports analysed for the WT1^+^, 19 were found to be positive in both blood and tissue; whereas 2 were WT1^+^ in blood only and one was negative in blood but positive in tissue. All tissue samples were CK7 positive, whereas 14/15 blood samples were CK^+^ as well. Therefore, there is good correlation between histopathological findings and imaging flow-cytometry.

### 3.4. NACT, post-PDS, and Relapse Cohorts

The numbers of CK^+^ or WT1^+^ cells per mL of blood from patients in each of the three groups: NACT (n = 13 for CK^+^ and n = 11 for WT1^+^) versus post-PDS (n = 9 for both CK^+^ and WT1^+^) versus Relapse (n = 15 for CK^+^ and n = 7 for WT1^+^), are depicted in Figure 4E,F and compared to healthy volunteers (n = 39 for CK^+^ and n = 15 for WT1^+^).

Blood from patients with newly diagnosed stage IIIc/IV (and one stage II) ovarian cancer prior to any treatment (NACT) or following primary surgery only (post-PDS), contained significantly higher numbers of CK^+^/CD45^−^/DRAQ5™^+^ staining CCs as compared to healthy controls (*p* = 0.0049 and *p* = 0.006 respectively). A similar, albeit non-significant trend was evident when controls were compared to patients after relapse (Figure 4E). The average CK^+^/CD45^−^/DRAQ5™^+^ CCs detected per ml of blood were: 47 for controls, 263 for NACT, 1155 for post-PDS, and 421 for relapse.

When blood from patients was stained for WT1^+^/CD45^−^/DRAQ5™^+^, the average CCs detected per ml of blood were: 667 for controls, 4761 for NACT, 1718 for post-PDS, and 1548 for relapse. NACT patients had significantly higher numbers of WT1^+^/CD45^−^/DRAQ5™^+^ CCs compared to healthy controls (*p* = 0.0046). Notably, post-PDS patients had fewer WT1^+^ cells (mean 1718) than the NACT group (mean 4761; *p* = 0.08), possibly reflecting the substantial reduction in tumour mass at surgery (Figure 4F).

### 3.5. Correlation of CC with CA125

**Figure 5:**
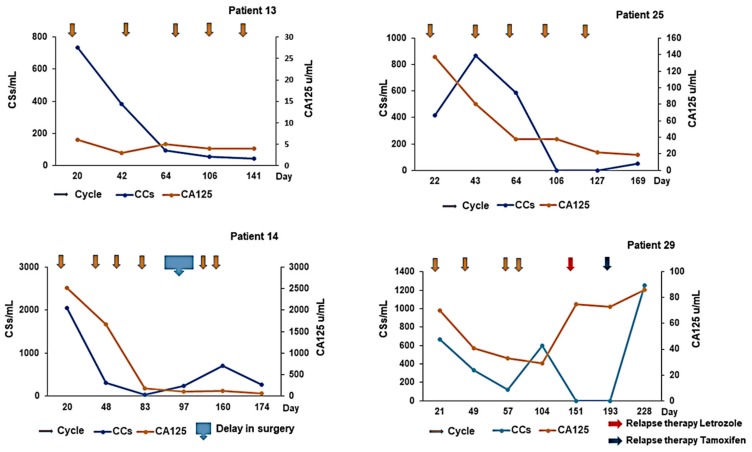
Depiction of CC levels (blue markers) and CA125 (orange markers) for 4 aEOC patients over time (measured in days), in a cohort treated with chemotherapy (arrows).

Overall, CCs (WT1^+^/CD45^−^/DRAQ5™^+^) varied in broad alignment with serum CA125 response (Figure 5); for example, patient 14 had a similar drop in CCs and CA125 levels following the first 3 treatments. However, when we performed correlation analysis (Pearson and Spearman) no apparent significance was reached. As it can be seen, patient 13 had adjuvant chemotherapy after primary surgery for stage IIB ovarian cancer and there was a good response that correlated with a drop in CC level, although no apparent change was noted for CA125 levels. A similar pattern was evident for patient 25, who received chemotherapy for first relapse ovarian cancer with a good clinical response. Patient 14, was a NACT patient that received 4 cycles of chemotherapy, followed by an unexpected 50-day delay prior to interval surgery. It is evident that although CA125 remained stable, CCs rose in line with disease relapse/progression during this period. Patient 29 is a post-PDS (post-surgery) patient showing early relapse during adjuvant chemotherapy at 130 days, which was preceded by a rise in CC level at 60–70 days. If we had known this, we may well have withheld her chemotherapy after 2–3 cycles due to it being ineffective. CC levels decreased dramatically following treatment with letrozole and subsequent tamoxifen (relapse therapy). A one month follow up demonstrated a rapid surge of CCs concomitant with the diagnosis of brain metastases and relatively early death of this patient.

### 3.6. Changes in Sizes of CCs

**Figure 6:**
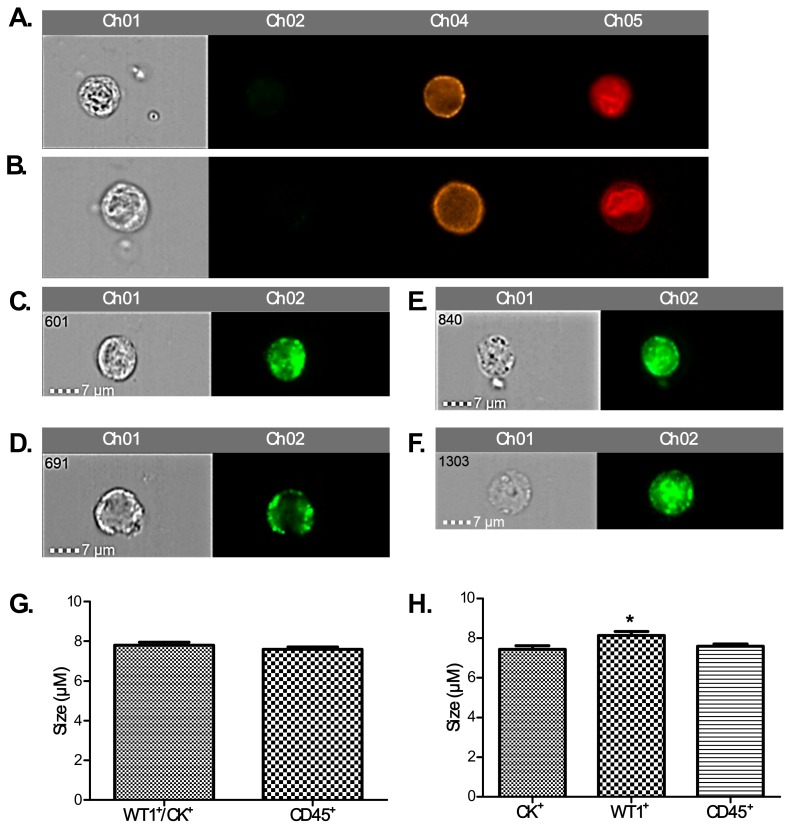
Different sizes of WBCs (**A**: 8µM; **B**: 10µM), CK^+^ CCs (**C**: 7µM, **D**: 8.5 µM), WT1^+^ CCs (**E**: 7µM; **F**: 8µM). (**G**): No apparent differences in size were detectable when combined CK^+^ and WT1^+^ CCs were measured and compared to controls. However, WT1^+^ CCs were larger compared to CK^+^ CCs; but not by a great margin (**H**; * *p* < 0.05).

We have assessed the average size of CCs in our cohort of aEOC patients using IDEAS software (magnification x60) for CCs (CK^+^/WT1^+^/CD45^−^) and WBCs (CK^−^/WT1^−^/CD45^+^). The average sizes were: NACT-CK^+^ 7.33 µM, NACT-WT1^+^ 8.13 µM, post-PDS-CK^+^ 7.70 µM, post-PDS-WT1^+^ 8.00 µM, Relapse-CK^+^ 7.50 µM, and Relapse-WT1^+^ 8.13 µM. No significant changes in these sub-groups were apparent. However, when CCs were grouped based on CK^+^ and WT1^+^ staining, there was a subtle but significant difference: the average size of CK^+^ was 7.46 µM and of WT1^+^ it was 8.09 µM (Figure 6).

### 3.7. Relapse Cohort-Looking beyond CCs

**Figure 7:**
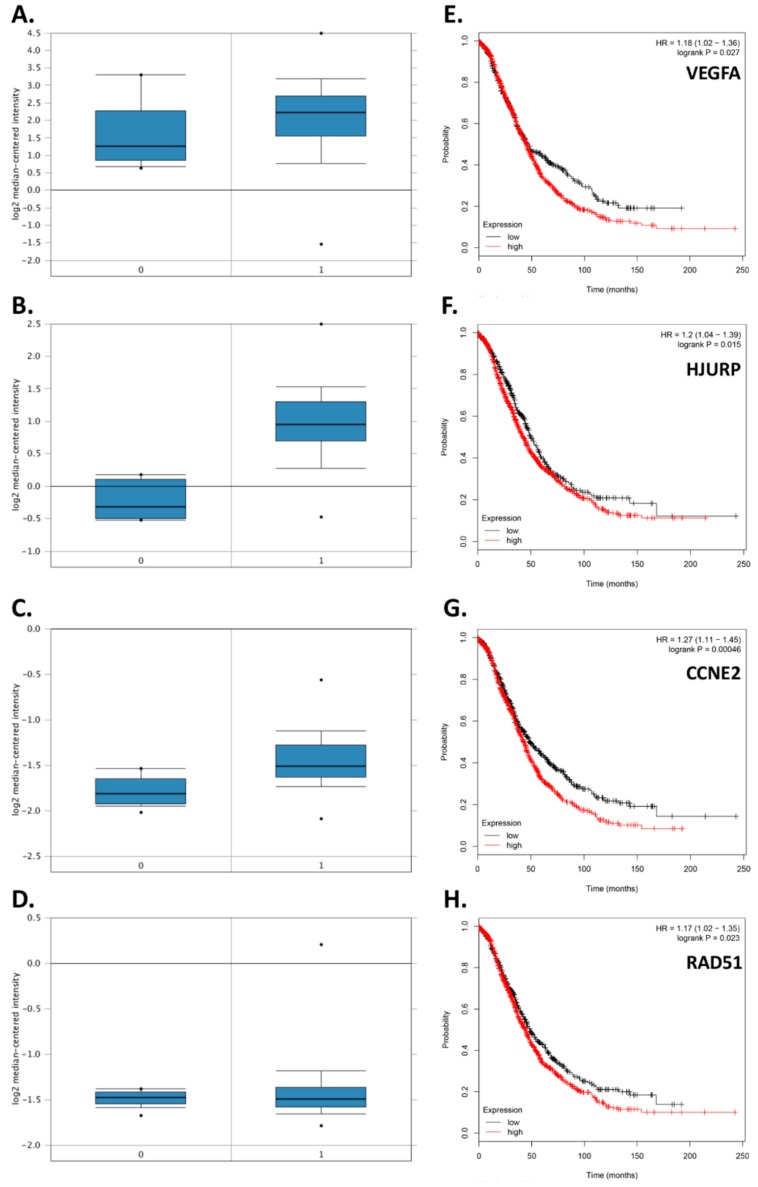
Oncomine analysis for VEGFA (**A**), HJURP (**B**), CCNE2 (**C**) and RAD51 (**D**) mRNA expression, using the Bonome ovarian dataset; Human Genome U133A Array (Normal benign controls (Lane 0) n = 10, and Ovarian Carcinoma (Lane 1) n = 185), demonstrate a significant upregulation for all four genes in the cancer cohort compared to controls: VEGFA (210512_s_at): (*p* = 1.24 × 10^−13^; fold change = 1.670), HJURP (218726_at): (*p* = 9.10 × 10^−10^; fold change = 2.347), CCNE2 (211814_s_at): (*p* = 5.80 × 10^−6^; fold change = 1.286) and RAD51 (205023_at): (*p* = 1.08 × 10^−4^; fold change = 1.475). Overall Survival (OS) plotted as a Kaplan Meier, shows poorer overall survival in the high expression group of OC patients for all 4 genes tested. More specifically: VEGFA (210512_s_at; (**E**): (*p* = 0.027, Low = 482 High = 1174), HJURP (218726_at; (**F**): (*p* = 0.015, Low = 459 High = 1197), CCNE2 (205034_at; (**G**): (*p* = 0.00046, Low = 686 High = 970), and RAD51 (205023_at; (**H**): (*p* = 0.023, Low = 554 High = 1102).

A 79-gene panel (TATAA Biocenter GrandPerformance assays) was used to explore whether RNA extracted from whole blood (i.e., including CCs and WBCs) would show a distinct molecular signature in a relapsed ovarian cancer cohort. For this part of the study, 25 samples were analysed from the 5 METRO-BIBF relapse aEOC patients (i.e., samples from sequential cycles of treatment for each of the 5 patients were also included) and compared to total blood RNA from 14 healthy controls (Figure S3). This revealed that in the METRO-BIBF patients 12 genes were up-regulated and 16 were down-regulated. Of the 12 upregulated genes VEGFA, HJURP, CCNE2, and RAD51 were also upregulated in the tissue of ovarian cancer patients according to in silico microarray data (Oncomine). Moreover, these genes were associated with worse overall survival (OS) in patients with aEOC (Kaplan Meier-Plot data) (Figure 7).

## 4. Discussion

In this study, rather than attempting to ‘enrich’ our samples for circulating tumour cells (CTCs), we chose to examine the whole liquid biopsy following red blood cell lysis. By co-staining with CK and WT1 markers, and CD45 to exclude WBCs, we show that there are abundant non-haematopoietic CCs (CK^+^, WT1^+^ and CD45^−^) in patients with aEOC compared to healthy female controls. Detection of these CCs is reproducible, is reliably undertaken and can be informative. Previous studies may have underestimated the importance of CCs in patients with stage III/IV aEOC, which seem to represent a window in the microenvironment in which ovarian CTCs flourish. We show here that the size of CCs in ovarian cancer is not significantly different from the size of the rarer non-haematological cells found in healthy controls, suggesting that efforts to identify ovarian CTCs based on size alone can be challenging and further studies needed to assess if treatments can impact on cell size. We have also shown, via a 79 gene panel, that 4 genes were found upregulated mirroring changes at tissue level, whereas EpCAM does not appear up-regulated in relapsed aEOC patients. Hence the important EMT derived CTC-population is also likely to be missed by currently used techniques involving EpCAM reliant enrichment [11].

A strength of flow cytometry is the ability to detect heterogeneity within a patient’s “CC-rich” sample, including sub-populations such as tumour cells and those of haematopoetic origin. However, flow cytometric assays are at risk of over-estimating rare events through non-specific antibody binding. Another false positive problem relates to antibody binding to soluble antigen forms. To mitigate these, we used DRAQ5™ staining for nuclear material to exclude apoptotic cells, cell debris, and platelet aggregates. Repeated gating and fluorescence compensation strategies may also augment inter-laboratory variability. Ideally >1 cell/µL is required to fulfil flow cytometric criteria with a coefficient variation of 10% [12].

A further limiting factor includes inter and intra-patient variability, described as differences in patient traits, characteristics, genetics and/or pharmacokinetics (including in some instances exposure to environmental influences) [13]. As highlighted earlier, the exact mechanism of CC shedding in malignancy is not well understood [5] and composition of CCs may differ from patient to patient, possibly in response to different biological and physiological states. Despite these potential limitations, we have shown that there are significantly lower numbers of CCs in patients with relapsed aEOC compared to newly presenting and immediately post-operative patients. In general, relapsed patients are not as systemically unwell as those at presentation, probably reflecting more timely interventions when we are already aware of the likelihood of relapse. Exploring the composition of the CCs in larger relapse patient cohorts is required to determine the relevance of this finding.

We chose to explore the presence/absence of the Wilm’s Tumour protein, WT1 in CCs as nuclear/cytoplasmic expression of WT1 has 96% specificity and 100% sensitivity as a marker in serous tubal, ovarian, peritoneal cancer [14,15]. To the best of our knowledge, no other reports detail WT1 expression on CCs in ovarian cancer and its potential to be a biomarker candidate for the identification of ovarian cancer CTCs. The median number of WT1^+^ CCs in our patient cohorts was 2914 per mL whole blood, which is significantly more than the median of 667 per mL found in the healthy volunteers. Most patients in this study had confirmed WT1 positive tissue diagnoses of serous ovarian, tubal, or primary peritoneal cancer. Surprisingly, in two young female volunteers, we detected ≥4000 WT1^+^ CCs/mL which, however, is consistent with the presence of circulating endothelial cells found in females during the menstrual phase [16]. Indeed, in our study, these two controls with high detectable levels WT1 were of menstrual age (31 years). The average age of all healthy volunteers was 40 years (range 21–64 years), with 7 of them not having any WT1^+^ cells detected. Future studies using a larger age-matched control cohort will provide a better insight into how reliable this may be in premenopausal women in terms of indicating disease burden.

We found a significantly higher number of cytokeratin-positive CCs amongst our patient population compared to healthy controls. Techniques for concomitant membrane and nuclear staining are not sufficiently optimised yet to show how many of these are also WT1+ to confirm that these are tumour cells. We postulate that a proportion of them might be circulating endothelial cells (CECs). The presence of CECs has been recognised as a potential biomarker of vascular damage in many cancers including ovarian (500 CECs/mL) and renal cell (800 CECs/mL) cancers [17,18,19]. Further work is on-going to confirm/refute this utilizing specific antibodies for endothelial cells (e.g., CD106, CD105, CD34, CD146). Preliminary evidence suggests presence of CD34^+^ (marker for CECs) cells in the blood of ovarian cancer patients (Figure S4). Another reason for the high numbers of CCs described in this study might be the absence of EPCAM enrichment, allows inclusion of cancer cells undergoing epithelial–mesenchymal transition (EMT), and not just those with pure epithelial phenotype.

EMT is a fundamental developmental process during which epithelial cells lose their polarity and junctional architecture to become mesenchymal cells that are motile and contributes in tumour metastasis [20]. High grade serous ovarian cancer is believed to originate in the fimbrial ends of the fallopian tube where cells express a mesenchymal phenotype [21]. E-cadherin is highly expressed in primary ovarian carcinomas, but this is lost in the advanced stages; a hallmark of EMT. This is suggestive of EMT facilitating the invasive ovarian cancer phenotype [22]. In a more recent study, the authors argue that EMT is not complete in epithelial ovarian cancer and tumors can retain both phenotypes [23]. In terms of EMT events recorded in liquid biopsies, breast cancer CTCs express mesenchymal and epithelial markers, but mesenchymal cells are highly enriched in CTCs and are associated with disease progression [24]. To the best of our knowledge, there is no data relating to an EMT phenotype amongst CTCs of ovarian / fallopian tube origin, however during EMT transition there is a decrease in the expression of cytokeratins in ovarian cancer cells [25]. Therefore, we hypothesised that the difference in staining found here could be also attributed to ovarian CCs undergoing EMT. In a proof of principle experiment using SKOV3 cells (which exhibit an intermediate mesenchymal phenotype) and PEO1 cells (which exhibit an epithelial phenotype); we show that more PEO1 than SKOV3 cells stained positive with CK.

Although, generally less sensitive than circulating-free DNA (cfDNA) [26,27] whole CC analysis can be performed at DNA, RNA (mRNA or micro-RNA) and protein level, providing further insight into the biology and microenvironment of cancer metastases. Contamination of CTCs with other non-haematopoietic CCs, is little different to examining solid tumour tissue where cancer cells are within a microenvironment of normal and reactive stromal tissue. Our data from whole blood provides novel insights into the differential expression of genes from liquid biopsies but also poses certain limitations. For example, WBCs expression patterns vary with both inflammatory and nutritional states which are difficult to predict to allow accurate assessment of changing CC phenotypes. Future studies should concentrate on assessing gene expression of CCs only. However, current methods rely heavily on EPCAM dependent isolation techniques which will miss cells undergoing EMT transition. Future studies could capitalise on data available from databanks, although the current bioinformatic indexes (e.g., Oncomine, The Cancer Genome Atlas; TCGA) rely mostly on microarray/RNAseq data from tissue rather than from liquid biopsies. Exploration of the CC signatures of larger cohorts of aEOC patients, and correlation with paired diagnostic tissue, is planned to identify changes which will hopefully yield alternative therapeutic strategies or targets that can be harnessed for prognostic purposes. We suggest that this simple method of extracting CC-rich information has the potential for exploring part of the circulating microenvironment within the remit of nearside patient testing and rapid responses required for 21st century treatment.

## Supplementary Materials

The following are available online at https://www.mdpi.com/2073-4409/8/7/732/s1, Figure S1: Proof-of-principle experiments using OC cell lines, Figure S2: changes in CK and WT1 staining depending on the epithelial/mesenchymal status, Figure S3: Gene expression data from whole blood, Figure S4: CD34^+^ (marker for CECs) cells in samples from two aEOC patients.
