# Supplementary material for: Detection of Abundant Non-Haematopoietic Circulating Cancer-Related Cells in Patients with Advanced Epithelial Ovarian Cancer
Source: Cells. 2019 Jul 17;8(7):732. doi: 10.3390/cells8070732 (PMC6678489; doi:10.3390/cells8070732)
Supplement: Supplementary file 1 [file cells-08-00732-s001.pdf]

Supplementary Figures

A

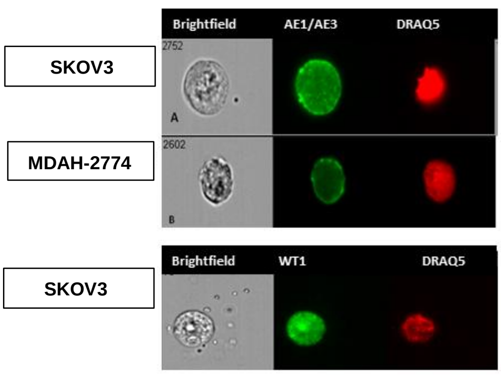

B

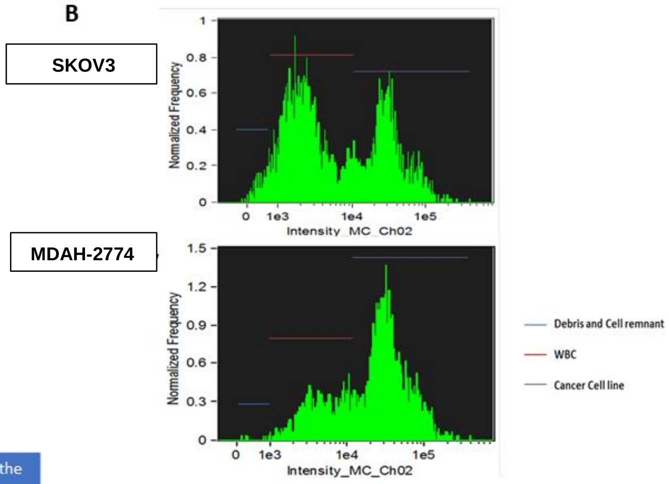

C

Total cells spiked Into 1 ml of blood	~ Retrieval of EpCAM^{+} stained cells	~ Retrieval of AE1AE3 (CK^{+}) stained cells	% difference in the retrieval
200,000	3.58% (7,168)	13.8% (27,520)	+283.9%
20,000	7.12% (1,421)	55.8% (11,169)	+686.0%
2000	10.7% (213)	28.1% (561)	+163.4%
200	32.5% (65)	48.5% (97)	+49.23%
20	20% (8)	60% (12)	+50%

Figure S1. Panel A: Images show positive AE1/AE3 staining of SKOV3 & MDAH-2774 cells. Positive AE1/AE3 staining, is signified by a deep green halo around cells cytoplasm, and positive DRAQ5 (red) staining of nucleus; 40 x magnification. **Panel B:** Separation of the captured cell populations, was measured by intensity of AF488 staining of the AE1/AE3 antibody in channel 2. The 3 populations highlighted in the charts identified based on staining pattern as labelled (blue for cell debris, red for white blood cells (WBC), and purple for the cell line). **Panel C:** Retrieval of cells from spiking experiments using EpCAM^{+} and CK^{+}.

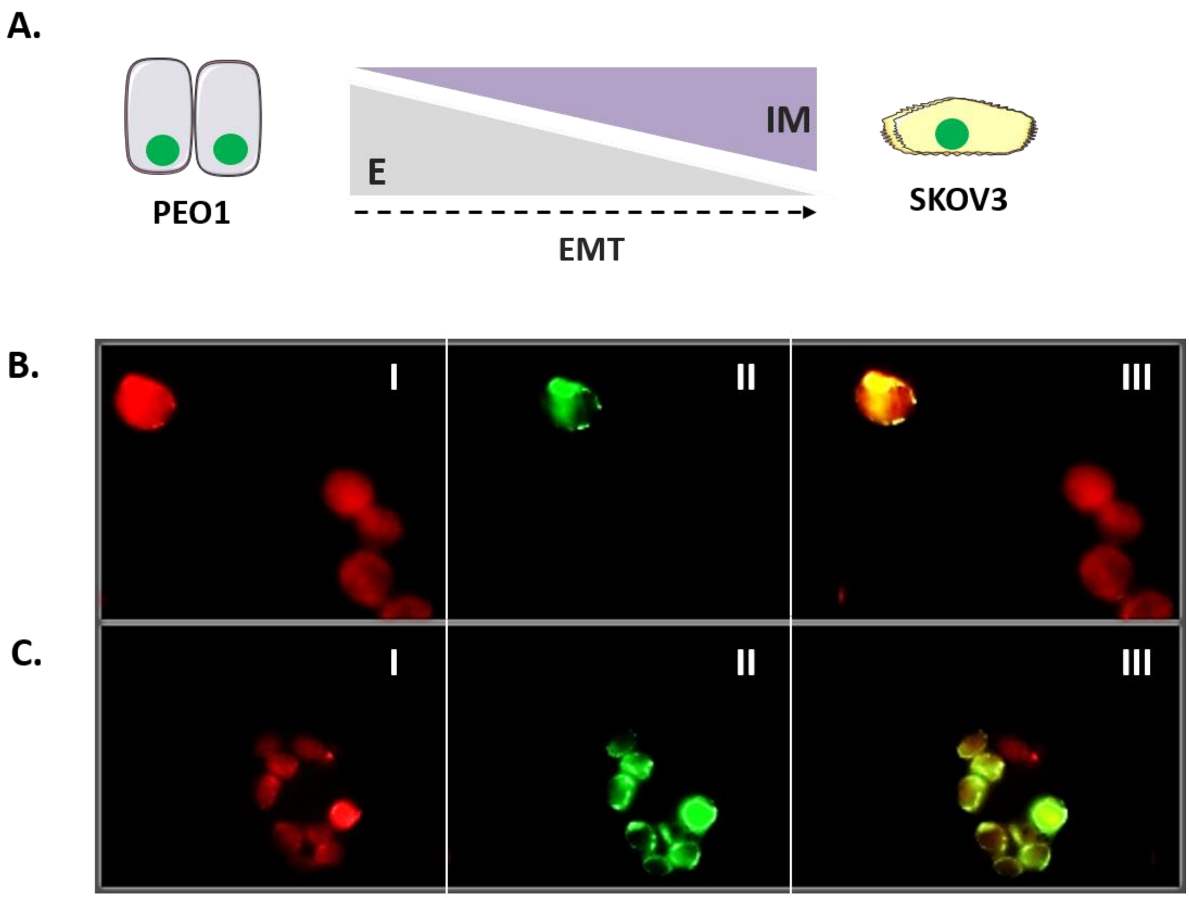

Figure S2. Panel A: Diagrammatic representation of EMT dynamics between SKOV3 and PEO1. SKOV3 cells **(B)** and PEO1 cell **(C)** stained with WT1 (red; I), AE1/AE3 (green; II) and merged images (III).

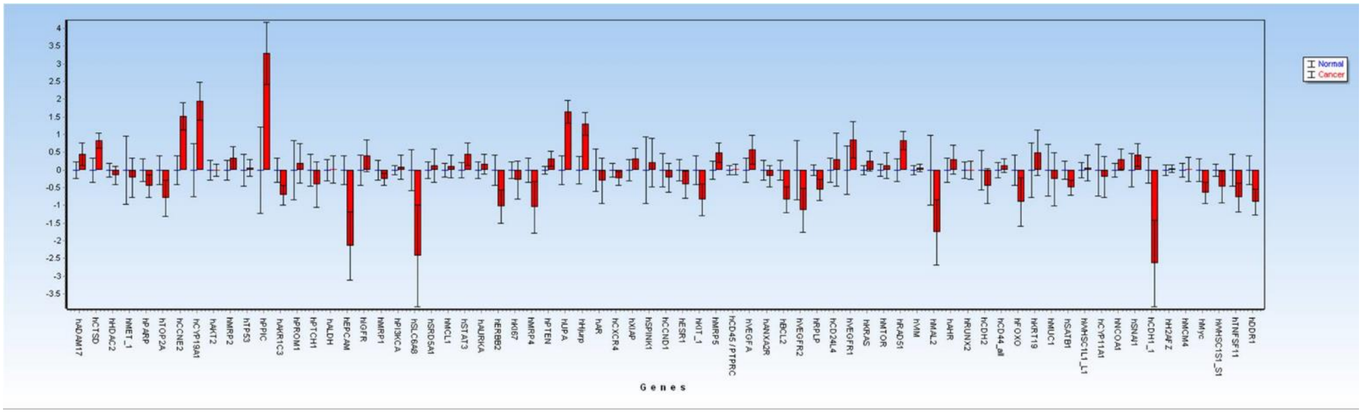

Figure S3. Gene expression of ovarian cancer blood samples compared to controls. Data generated from ovarian cancer panels is plotted as a box plot chart for both ovarian cancer and controls. Expression of 28 genes found to be significantly (p-value<0.05) up or down-regulated in cancer cohort compared to controls.

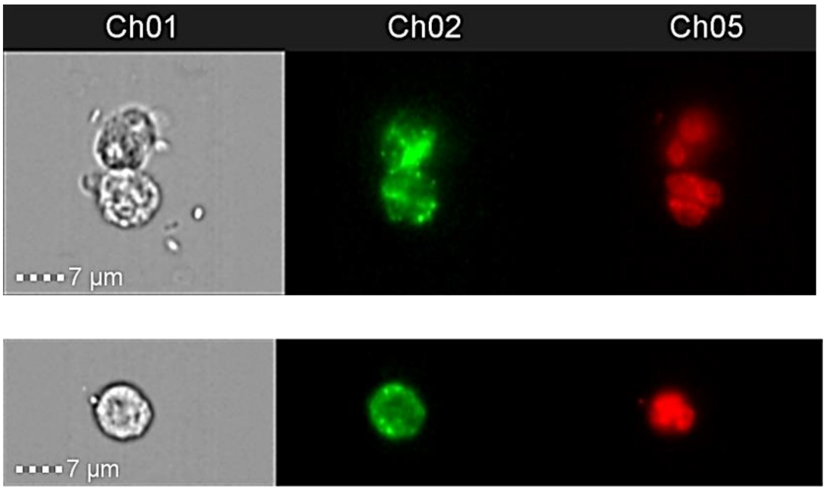

Figure S4. Presence of CD34+ (marker for CECs) cells in samples from two aEOC patients. Ch01: Brightfield; Ch02: CD34^{+}; Ch05: DRAQ5.
